# The Effect of Technology-Mediated Diabetes Prevention Interventions on Weight: A Meta-Analysis

**DOI:** 10.2196/jmir.4709

**Published:** 2017-03-27

**Authors:** Rachel R Bian, Gretchen A Piatt, Ananda Sen, Melissa A Plegue, Mariana L De Michele, Dina Hafez, Christina M Czuhajewski, Lorraine R Buis, Neal Kaufman, Caroline R Richardson

**Affiliations:** ^1^ University of Michigan Medical School Ann Arbor, MI United States; ^2^ Department of Learning Health Sciences University of Michigan Ann Arbor, MI United States; ^3^ University of Michigan Department of Family Medicine Ann Arbor, MI United States; ^4^ University of Michigan Department of Biostatistics, School of Public Health Ann Arbor, MI United States; ^5^ University of Michigan Department of Internal Medicine Ann Arbor, MI United States; ^6^ University of Michigan School of Information Ann Arbor, MI United States; ^7^ Geffen School of Medicine and Fielding School of Public Health Departments of Pediatrics and Public Health University of California Los Angeles, CA United States

**Keywords:** diabetes mellitus, type 2, weight reduction programs, technology, meta-analysis, prediabetic state

## Abstract

**Background:**

Lifestyle interventions targeting weight loss, such as those delivered through the Diabetes Prevention Program, reduce the risk of developing type 2 diabetes. Technology-mediated interventions may be an option to help overcome barriers to program delivery, and to disseminate diabetes prevention programs on a larger scale.

**Objective:**

We conducted a meta-analysis to evaluate the effect of such technology-mediated interventions on weight loss.

**Methods:**

In this meta-analysis, six databases were searched to identify studies reporting weight change that used technology to mediate diet and exercise interventions, and targeted individuals at high risk for developing type 2 diabetes. Studies published between January 1, 2002 and August 4, 2016 were included.

**Results:**

The search identified 1196 citations. Of those, 15 studies met the inclusion criteria and evaluated 18 technology-mediated intervention arms delivered to a total of 2774 participants. Study duration ranged from 12 weeks to 2 years. A random-effects meta-analysis showed a pooled weight loss effect of 3.76 kilograms (95% CI 2.8-4.7; *P*<.001) for the interventions. Several studies also reported improved glycemic control following the intervention. The small sample sizes and heterogeneity of the trials precluded an evaluation of which technology-mediated intervention method was most efficacious.

**Conclusions:**

Technology-mediated diabetes prevention programs can result in clinically significant amounts of weight loss, as well as improvements in glycaemia in patients with prediabetes. Due to their potential for large-scale implementation, these interventions will play an important role in the dissemination of diabetes prevention programs.

## Introduction

Over 29 million Americans (approximately 9% of the US population) have diabetes, and an additional 86 million Americans have prediabetes, an asymptomatic condition associated with an increased risk of developing type 2 diabetes [1]. In 2002, the landmark Diabetes Prevention Program (DPP) demonstrated that improved diet, regular exercise, and weight loss lead to a 58% reduction in the 3-year incidence of type 2 diabetes, compared to a placebo control arm, in patients at risk for the disease [2]. Importantly, this reduction was sustained over the long-term; specifically, 10-year follow-up analysis demonstrated the incidence of type 2 diabetes in the lifestyle intervention group was reduced by 34% compared with placebo [3].

The remarkable success of the DPP generated nationwide efforts to translate the results of the original trial intervention into practice [4,5]. The National Diabetes Prevention Program (NDPP) was established in 2010 as a congressionally-authorized initiative to support the dissemination of diabetes prevention programs across the United States [6]. NDPP-recognized diabetes prevention programs consist of 16 weekly sessions (core phase) followed by 6 monthly sessions (postcore phase) delivered by a trained lifestyle coach following the curriculum of the original DPP.

Systematic review of in-person group-based DPP programs demonstrates that they can effectively promote weight loss [7]. Although community-based DPP translations are more accessible, scalable, and financially sustainable than one-on-one interventions used during the original DPP study [4,6], there are barriers to participation. These barriers include transportation, distance, work schedules, aversion to group settings, and child care needs [8-11]. To address these issues, several pilot studies have used technology-mediated interventions to promote weight loss in participants at risk for type 2 diabetes. To date, a systematic review of these studies has not been performed.

The primary purpose of this meta-analysis is to assess the effect of technology-mediated lifestyle interventions on weight loss in those at risk for developing type 2 diabetes. We also discuss reported glycemic changes associated with these interventions.

## Methods

### Study Selection

We followed the Preferred Reporting Items for Systematic Reviews and Meta-Analyses statement guidelines for conducting and reporting this meta-analysis (Multimedia Appendix 1) [12]. We examined studies evaluating interventions that used technology to disseminate diet and exercise lifestyle programs, with the aim to achieve weight loss and improve glycemic control in adult patients with prediabetes. A systematic review was performed on the literature published between January 1, 2002 and August 4, 2016. Only studies published after the 2002 DPP study were included. We searched 6 databases to identify relevant studies, including PubMed, EMBASE, SportDiscus, CINAHL, PyschINFO, and Web of Science. Search terms to assess lifestyle intervention and use of technology were used, including the combination of MeSH and Emtree headings and subheadings, free-text keywords, and study design filters (eg, prediabetic state, weight loss, weight reduction programs, prediabet*, diabetes prevent*, telemedicine, telephone, web, technolog*, randomized controlled trial, and controlled clinical trial). We manually searched reference lists of review articles, and experts in the field were contacted to include all possible studies. Studies targeting individuals younger than 18 years of age, pregnant patients, or patients with a diagnosis of type 2 diabetes mellitus were excluded (see Multimedia Appendix 2 for search strategy).

Article titles and abstracts were screened to determine relevance and possible inclusion in the study. Full texts of the resulting articles were then read to determine eligibility based on the inclusion criteria. Authors were contacted directly to request missing weight change data, or clarify intervention methods and participant criteria when necessary.

Studies considered for inclusion had to satisfy three criteria. First, the primary objective of the study was to deliver diet and exercise lifestyle interventions using technology (digital versatile disc [DVD], computer-based program, phone, or text messaging) with the aim to achieve weight loss. Second, the study had to target patients with a diagnosis of prediabetes, or a body mass index (BMI) >24 kilograms per meter squared (kg/m^2^; or >22 kg/m^2^ if Asian) and at least one additional risk factor for diabetes (prior gestational diabetes, central adiposity, or metabolic syndrome); these criteria were based on those employed in the NDPP criteria [13]. Third, the study had to be either a randomized controlled trial (RCT) or prospective cohort study—with or without comparison groups—published in an academic journal and reported in English.

### Outcomes

The primary outcome was absolute weight change following the intervention. Glycemic changes (as measured by changes in oral glucose tolerance test results, fasting blood glucose levels, hemoglobin A1c levels, prediabetes prevalence, or incidence of diabetes over the intervention period) were also reported, if available.

Studies that included a core intervention phase, as well as a postcore maintenance phase, had data extracted and used for this analysis directly after the core phase was completed. This approach served to reduce heterogeneity between studies that did and did not include a maintenance phase. We assessed absolute weight change effect of technology-mediated interventions, and compared results between those interventions modeled on the DPP with those using a different curriculum. We also examined the influence of intervention duration on weight change. Average percent weight change was also reported, if available. Program attrition was also assessed by comparing the number of enrolled participants with the number of program completers.

### Data Analyses

Weight change outcomes in the core phase of each intervention were assessed using a meta-analysis. These outcomes were either directly reported in the study results, calculated by determining the within-person difference between reported weights before and after the intervention, or obtained from the authors. The focus of our study was to observe the effect of technology-mediated interventions on weight change, and since the control groups across the different papers were significantly disparate, we excluded the data reported for control arms and only extracted data from the groups receiving an intervention delivered by technology. Each treatment group (or cohort) was analyzed as one pre/postintervention study. All weights were converted to kg units. Using the standard deviation (SD) of within-person weight change outcomes was necessary for calculating the relative study-influences for the meta-analysis. Studies that did not directly report this value had SDs calculated using either the *P*-value or the CI associated with average weight change.

Heterogeneity between studies was assessed by Cochran’s Q statistic, which is the weighted sum of squared deviations of the study-specific estimates from the overall one, and is distributed approximately as a chi-squared random variable with k -1 degrees of freedom (k being the number of studies in the analysis) [14]. We further measured the *I*^2^ statistic, defined as *I*^2^=100%*(Q-k+1)/Q, that quantifies the proportion of heterogeneity in the trial results beyond chance. Higher *I*^2^ is indicative of greater heterogeneity. Based on the observation of significant heterogeneity across studies, study estimates were pooled using a random-effects model that allowed for some random variability between studies, as well as sampling error. Publication bias between studies was assessed visually using a funnel plot. The visual effect is supplemented by the more formal Egger’s test [15], which is essentially a significance test of intercept in a weighted least-squares fit of study-specific standardized effect on precision (reciprocal of standard error). A significant intercept is indicative of publication bias. Rosenthal’s fail-safe N, based on the aggregated standardized effect size, was used to estimate the number of additional (potentially unpublished) studies required to convert a significant result into a nonsignificant one. A fail-safe N>5k+10 is indicative of lack of potential publication bias [16]. An influence analysis was carried out to assess the influence of each study by recalculating the pooled estimate after deleting the study.

The included studies were of varied types—ranging from observational to blinded and unblinded randomized trials (with or without control)—making a fixed protocol for quality scoring impractical. Instead we performed an indirect quality-adjusted analysis via a meta-regression, adjusting for study duration, examining whether the analysis used intention-to-treat methods, and whether the intervention incorporated DPP material. Statistical analyses were carried out in STATA 13 (StataCorp LP, College Station, Texas) and R 3.3.0 (R Foundation for Statistical Computing, Vienna, Austria).

## Results

### Results of Systematic Literature Search

**Figure 1:**
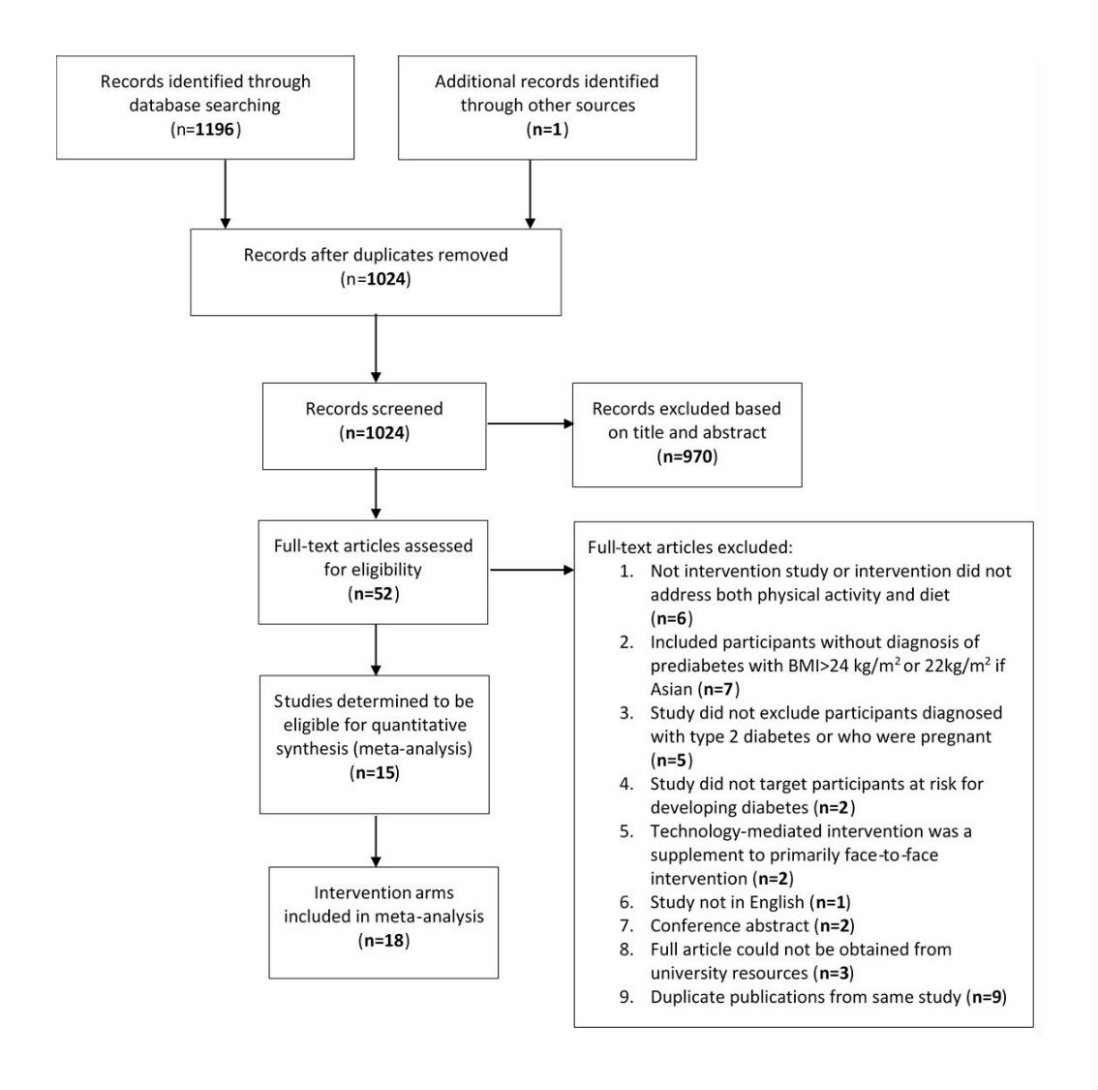
Flowchart showing results of systematic search strategy and selection process. BMI: body mass index.

After eliminating duplicates, a total of 1024 publications were identified through the databases; one additional publication was identified from searching reference lists and through consultation with experts. Each publication was screened by title and abstract. The resulting 52 publications were reviewed in full, 37 of which were excluded because they did not meet inclusion criteria. The final systematic review included 15 publications that reported outcomes for a total of 18 intervention arms (Figure 1).

### Study Characteristics

Of the 15 studies included: 6 were RCTs in which the technology-mediated treatment arm was compared to standard care or a face-to-face intervention; 2 included control arms but assigned treatment conditions by community and without randomization; 3 randomly assigned participants to different technology-mediated intervention arms; 1 included parallel interventions arms assigned by community and without randomization; 2 were prospective cohort studies; and 1 allowed participants to self-select into the treatment arm. Multimedia Appendix 3 describes each cohort, and Table 1 shows key outcomes.

**Table 1:** Weight change outcomes. DVD: digital versatile disk; kg: kilogram; NA: not available or not applicable; SD: standard deviation.

Study Cohort (Year): Intervention	Attrition	Level of Utilization	Mean weight change, kg (SD)	% weight change (SD)
Aguiar et al (2016): DVD [17]	19%	NA	-4.98 (4.2)^a^	-4.85 (4.1)
Block et al (2015): Interactive voice response, email, text message, mobile app [18]	14%	After 6 months, intervention participants interacted with online program in a median of 17 of 24 weeks.	-3. 1 (3.6)^a^	-3.60 (NA)
Sakane et al (2015): Telephone [19]	18%	Mean responses to telephone calls during 1-year period: 2.8 (SD 0.6; Group A), 5.2 (SD 1.9; Group B), 8.2 (SD 3.5; Group C)	-1.1 (3.4)^a^	NA
Cha et al (2014): Internet and telephone [20]	13%	NA	-2.9 (4.3)	NA
Nicklas et al (2014): Internet [21]	11%	Median 9 videos watched	-2.6 (5.5)^a^, at 6 months	NA
Sepah et al (2014): Internet [22]	15%	85% (187/220) completed at least 4 of the 16 core lessons	-5.0 (3.6)	-5.0 (NA)
Betzlbacher et al (2013): Telephone [23]	0%	All calls completed	-3.3 (4.3)	-3.7 (NA)
Ma et al (2013): DVD and email [24]	10%	NA	-4.5 (7.2)^a^	-4.9 (7.2)
Piatt et al (2013): DVD [25]	43%	Average attendance for DVD debriefing sessions 2.9/4	-5.5 (4.0)^a^	-5.7 (4.0)
Piatt et al (2013): Internet and e-counseling [25]	57%	Average 6.8 of 12 videos viewed	-6.2 (5.1)^a^	-6.3 (4.5)
Ramachandran et al (2013): Text message [26]	4%	Average number of text messages dropped from 18 to 12 messages a month	-0.1 (2.7)	NA
Weinstock et al (2013): Individual telephone [27]	43%	Average 9 of 16 sessions attended across 2 intervention cohorts	-4.6 (17.6)	-4.2 (16.9)
Weinstock et al (2013): Group telephone [27]	38%	Average 9 of 16 sessions attended across 2 intervention cohorts	-4.9 (17.7)	-4.5 (20.3)
Kramer et al (2010): DVD [28]	14%	Average 10.2 of 12 calls completed	-5.4 (5.2)^a^	-5.6 (NA)
Vadheim et al (2010): Video conference [29]	12%	Average 14.2 of 16 weeks of participation	-6.7 (3.7)	NA
Estabrooks and Smith-Ray (2008): Interactive voice response [30]	28%	10% did not complete any calls	-2.2 (2.7)	-2.6 (3.1)
Tate et al (2003): Internet only [31]	15%	NA	-2.0 (5.7)^a^	-2.2 (NA)
Tate et al (2003): Internet and e-counseling [31]	17%	NA	-4.4 (6.2)^a^	-4.8 (NA)

^a^results reported for intention-to-treat analysis

### Participant Characteristics

A total of 2774 participants were enrolled in the technology-mediated interventions, of whom 2247 had follow-up data included in the final meta-analysis. Averaged across studies, enrolled participants were 49 years old and had a starting BMI of 29 (excluding 1 study that did not report baseline BMI [30]). Thirty-nine percent of the participants were female (excluding 1 study that did not report the gender composition of the intervention cohort [28]), and 34% were white (excluding 4 studies that did not report the number of white participants in the intervention cohorts [28,29]). Four studies were conducted outside of the United States [17,19,26,32] and 2 were undertaken in rural communities [25,29].

### Treatment Characteristics

The duration of the interventions ranged from 12 weeks to 2 years. Half of the studies were modeled on the DPP [18,21,22,24,25,27-29]. The technologies employed by the 18 intervention arms included DVDs and e-videos [17,21,24,25,28], Web-based resources [18,20-22,31], videoconferencing [29], telephone (individual and conference calls) [19,20,25,27,28], interactive voice response [18,30], text messages [26], e-counseling [21,24,25,31], email [18,24,25,31], and online group forums [22]. Supplementary print materials, such as diet and physical activity log books [17,19,23,25,28,29] and in-person group DPP [25,29], were also utilized. The lessons and messages delivered via the technology-enabled interventions centered on educating participants on how to achieve a healthy diet and exercise to reduce the risk of type 2 diabetes, and enabling behavioral changes through goal setting, self-monitoring, and logging of diet and physical activity. Video, text message, or Web-based lessons often introduced diet and physical activity concepts, while the personalized or automated phone, text message, and email messages would reinforce concepts, goals, and self-monitoring behavior.

### Publication Bias

**Figure 2:**
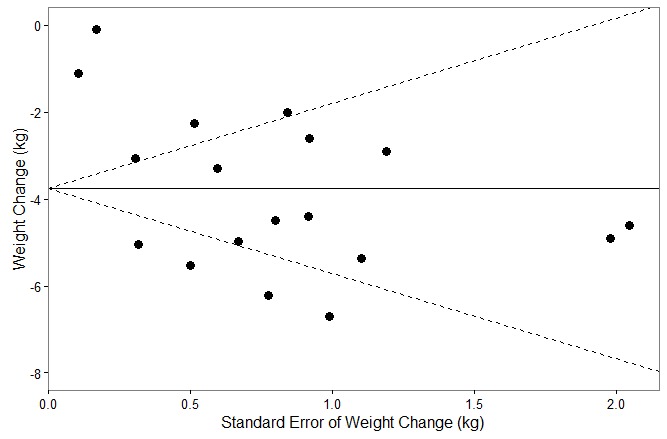
Funnel plot for publication bias analysis.

The funnel plot in Figure 2 was found to be statistically significant (*P*=.002) using Egger’s test, indicating a potential for publication bias. However, Rosenthal’s fail-safe N was above 4000 (more than 40 times the 5k+10 threshold) suggesting the potential threat from such bias to be quite small. For this reason, no corrective action was undertaken.

### Change in Weight

**Figure 3:**
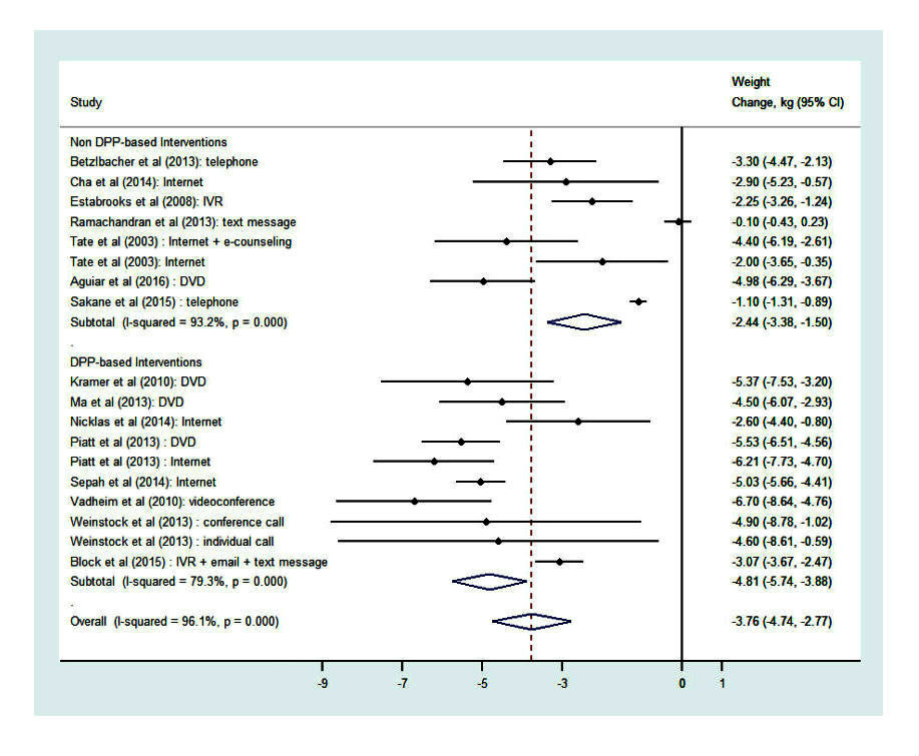
Forest plot of weight change from random-effects meta-analysis outcomes. DPP: Diabetes Prevention Program; DVD: digital versatile disk; IVR: interactive voice response.

**Figure 4:**
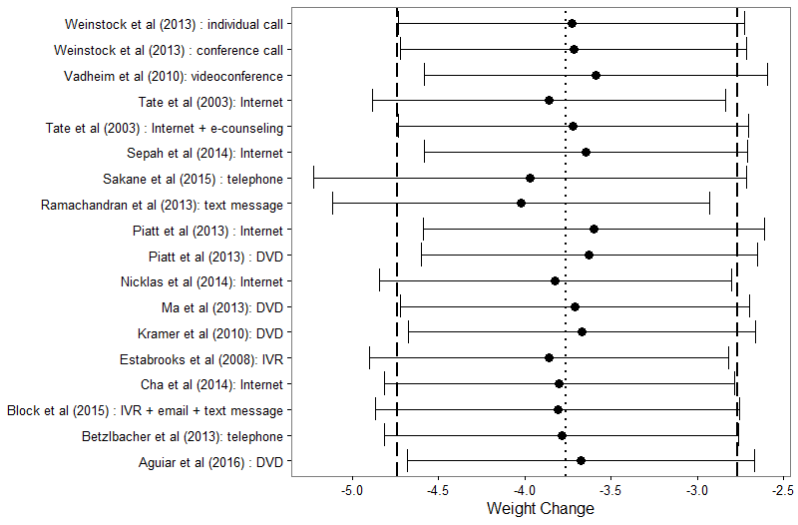
Influence plot of random-effects meta-analysis. DVD: digital versatile disk; IVR: interactive voice response.

Given significant heterogeneity across studies (I^2^=96.1%, *P*<.001), a random-effects meta-analysis of the change in weight was performed utilizing 18 cohorts from the 15 studies. Figure 3 displays the change in weight and CIs for each intervention, stratified by whether the intervention was modeled from the DPP. Figure 3 also presents a pooled estimate of mean weight change using a random-effects model combining data from all 18 cohorts. These results demonstrate that technology-based interventions are effective at decreasing weight by an average of 3.76 kg (95% CI 2.8-4.7; *P*<.001). The DPP-based interventions resulted in marginally higher (*P*=.074) average weight loss (mean 4.81 kg, 95% CI 3.9-5.7) than non-DPP interventions (mean 2.44 kg, 95% CI 1.5-3.4). The contribution of each study to the overall effect ranged from 3.2-6.6%. Furthermore, the influence plot in Figure 4 indicates that no single point had exceptional influence, and the exclusion of any single cohort did not appreciably change the results of the overall estimate.

### Change in Glycaemia and Follow-Up Weight Outcomes

Eight studies reported a change in glycaemia over the course of the intervention [17,18,20,22-25,28]. Multimedia Appendix 4 shows the particular measure(s) used—oral glucose tolerance test, fasting blood glucose, and hemoglobin A1c—as well as baseline and postintervention mean values. Multimedia Appendix 4 also shows the reduction in prediabetes prevalence among participants. All 5 of the studies that reported baseline and postintervention prediabetes prevalence measurements reported decreases in prevalence [17,18,20,23,25]. Of the 4 cohorts reporting conversion from prediabetes to diabetes during the intervention period, rates ranged from 0-18% [21,23,24,26]. The 2 cohorts with the longest intervention durations reported the largest conversion rates [23,26].

Multimedia Appendix 5 presents change in weight measured in follow-up or maintenance periods, which were documented 8 to 36 months after the initial postcore intervention measurements. Follow-up or maintenance period durations varied by study, ranging from 12 to 48 months after baseline measurements. Some cohorts followed their core interventions with less intensive and/or optional maintenance interventions [21,22,24,27,33], while others included follow-up measurements without postcore maintenance interventions [23,25,34,35]. The relationship between follow-up time and weight change was mixed, with some shorter time frames observing a larger change, and some longer time frames observing a smaller change.

## Discussion

This meta-analysis shows that technology-mediated interventions lead to clinically significant weight loss (mean 3.76 kg, 95% CI 2.8-4.7 kg; *P*<.001) in individuals at risk for diabetes. Additionally, as evidenced by our finding that 8 of 18 intervention arms [19,22,24,25,27,32-35] reported sustained weight loss outcomes at least one-year postintervention, the weight loss achieved through technology-mediated interventions may be sustainable. Moreover, the finding that several of the studies [17,18,20,23-25,28] reported improved glycaemia further supports the argument that these interventions are effective methods to prevent the development of diabetes.

Among American adults, there is widespread adoption of cell phones (92%) and smartphones (67%) [36]. These usage levels indicate that placing technology-mediated interventions in the hands of patients is becoming easier than ever. Moreover, from a clinical standpoint, we may soon live in a world where provider referrals to technology-mediated interventions to promote lifestyle and behavior change are commonplace. However, the marketplace is currently filled with a patchwork of technology-mediated solutions that vary widely in terms of quality, particularly in terms of health-related apps. This market is filled with products whose development often lacks professional content-expertise, theoretical underpinnings, and an appropriate evidence base to support use [37,38]. Given that we have demonstrated that quality technology-mediated interventions are effective at promoting and sustaining weight loss, more should be done to promote their use within clinical practice, so that consumers may succeed with high quality applications, as opposed to fail with poorly conceptualized, designed, and implemented tools.

Two design components that were of particular interest in this meta-analysis were: (1) studies based on the DPP curriculum, and (2) study duration. While not quite achieving statistical significance, there was a strong trend toward more weight loss in the intervention arms that were based on the DPP curriculum. Studies investigating the efficacy of technology-based interventions often rely on short-term follow-up, which is commonly seen as a limitation of the existing body of literature. Our analysis shows no effect of intervention duration on weight loss outcomes, which is not consistent with previously reported literature [7]. It is possible that this finding is due to the heterogeneity of the technologies utilized, or the fact that some interventions included maintenance phase components while others did not.

### Limitations

One limitation of our meta-analysis was the inability to compare intervention and control weight change results within the studies. This issue arose because only four studies were RCTs with a nontechnology control arm. To demonstrate whether technology-mediated interventions have comparable weight loss outcomes to in-person interventions, future studies are needed that directly compare these two cohorts. There was wide heterogeneity in intervention type, duration, population, and study attrition. Furthermore, none of the included studies had large sample sizes. Thus, conclusions could not be drawn about which method would be most efficacious. The variety of reported glycaemia changes limited our ability to perform a meta-analysis of these results. There was a fair amount of heterogeneity across the studies when examining study populations, as well as interventions used. This heterogeneity raises concerns for the statistical pooling of study results, but it strengthens the generalizability of the conclusions drawn from this systematic review, and provides important implications for the implementation of diabetes prevention programs.

Quality assessment was difficult, given the variety of study designs. Even in those studies that were RCTs, several standard quality criteria were not applicable. For example, complete blinding cannot be achieved within behavioral intervention studies. Instead, we chose three quality criteria that were relevant across studies (intention-to-treat analysis, intervention duration, and whether the study was modeled after the DPP) and included them as independent predictors in the meta-regression.

### Future Directions

Those technology-mediated interventions that were modeled after the DPP tended to result in greater weight loss compared to the non-DPP modeled interventions, although this difference was not statistically significant at the .05 level. Further research is needed to test the hypothesis that weight loss outcomes can be optimized by incorporating the DPP curriculum.

Several studies investigated the effect of technology-mediated interventions on specific populations, such as younger populations or those in rural settings. Further investigation is needed using large methodologically sound comparative-effect research trials to determine which interventions are most efficacious in facilitating weight loss and glycemic improvement in specific demographic categories of participants at risk for developing diabetes. Such categories include specific age ranges, BMIs, and genders, as well as social, economic, and ethnic backgrounds. It has been shown that the success of a lifestyle intervention is largely affected by the participant’s ability to choose the intervention modality [25], but factors that make an intervention more efficacious among target populations need to be explored, especially as technology allows intervention delivery to be individualized. Future studies should also evaluate whether technology should be coupled with some degree of in-person contact.

Studies are needed to examine how accessible these interventions are in low-income urban populations. For technology-mediated diabetes prevention interventions to expand the reach of diabetes prevention to a greater number of individuals at high risk for developing diabetes, research is needed with respect to intervention cost and payment models.

The inconsistency between using fasting blood glucose and hemoglobin A1c to measure metabolic control made it difficult to determine the extent to which technology-mediated interventions affected glycemic improvement. Weight loss was instead used as a direct health outcome measure. Some interventions may not result in weight loss but may improve glycemic control. Ideally, studies would include both weight loss and glycemic control (which would be measured using both fasting glucose and hemoglobin A1c) as a standardized evaluation methodology.

While a few of the studies in this analysis compared different forms of technology, further analysis is required to understand the advantages that each technology contributes to intervention outcome. In addition, the efficacy of maintenance phase interventions needs to be adequately assessed by comparing the weight loss outcomes and glycaemia of those who choose to stay in maintenance phase and those who decide to drop out. Understanding of such factors could guide the establishment of technology-mediated interventions as a potential correlate to the NDPP’s current program.

### Conclusion

Our meta-analysis showed that technology-mediated diabetes prevention interventions resulted in weight loss and lead to significant improvements in glycaemia. These results suggest that technology-mediated interventions could be an alternative to in-person diabetes prevention programs. The option of using technology-mediated delivery can potentially overcome barriers of access and allow expanded dissemination of such interventions.

## Appendix

Multimedia Appendix 1 Preferred Reporting Items for Systematic Reviews and Meta-Analyses checklist.

Multimedia Appendix 2 Search strategy.

Multimedia Appendix 3 Published studies of technology-mediated diabetes prevention interventions.

Multimedia Appendix 4 Change in glycaemia.

Multimedia Appendix 5 Follow-up weight outcomes.

## Abbreviations

BMI: body mass index
DPP: Diabetes Prevention Program
DVD: digital versatile disc
k: number of studies in the analysis
kg: kilogram
NDPP: National Diabetes Prevention Program
RCT: randomized controlled trial
SD: standard deviation
