# Supplementary material for: The Effect of Technology-Mediated Diabetes Prevention Interventions on Weight: A Meta-Analysis
Source: J Med Internet Res. 2017 Mar 27;19(3):e76. doi: 10.2196/jmir.4709 (PMC5387112; doi:10.2196/jmir.4709)
Supplement: Multimedia Appendix 2 [file jmir_v19i3e76_app2.pdf]

Appendix Search Strategy

Pubmed
#1 "Weight Loss"[Mesh]
#2 "Weight Reduction Programs"[Mesh]
#3 "weight"[Text Word]
#4 BMI[Text Word]
#5 "body mass index"[Text Word]
#6 OR / 1-5
#7 "Diabetes Mellitus, Type 2/prevention and control"[Mesh]
#8 "Group lifestyle balance"[Text Word]
#9 "Prediabetic State/prevention and control"[Mesh]
#10 "Prediabetic State/therapy"[Mesh]
#11 diabetes risk[Text Word]
#12 diabetes prevent*[Text Word]
#13 prediabet*[Text Word]
#14 OR / 7-13
#15 online[Text Word]
#16 internet[Text Word]
#17 virtual[Text Word]
#18 computer[Text Word]
#19 digital[Text Word]
#20 DVD[Text Word]
#21 video[Text Word]
#22 mobile[Text Word]
#23 telehealth[Text Word]
#24 technolog*[Text Word]
#25 telephone[Text Word]
#26 phone[Text Word]
#27 smartphone[Text Word]
#28 text messag*[Text Word]
#29 ehealth[Text Word]
#30 mhealth[Text Word]
#31 web[Text Word]
#32 OR / 15-31
#33 “Adolescent"[Mesh]
#34 "Child"[Mesh]
#35 33 OR 34
#34 6 AND 14 AND 33 NOT 35

Limits: English, publication date from 01/01/2002 to 08/04/2016

EMBASE, Ebsco (CINAHL, PsychInfo, SportDiscus)

(online OR internet OR virtual OR computer OR digital OR DVD OR video OR mobile OR telehealth OR technology OR telephone OR phone OR smartphone OR text message

OR text messaging OR ehealth OR mhealth OR web) AND (weight OR BMI OR "body mass index") AND (diabetes risk reduc* OR diabetes prevent* OR prediabet*) limited to after 2002, limited to adults, etc

#1 online
#2 internet
#3 virtual
#4 computer
#5 digital
#6 DVD
#7 video
#8 mobile
#9 telehealth
#10 technology
#11 telephone
#12 phone
#13 smartphone
#14 text messag*
#15 web
#16 ehealth
#17 mhealth
#18 OR / 1-17
#19 “body mass index”
#20 weight
#21 BMI
#22 OR / 19-21
#23 diabetes risk reduc*
#24 diabetes prevent*
#25 prediabet*
#26 OR / 23-25
#27 18 AND 22 AND 26

Limits: English, adults, Publication Date from 01/01/2002 to 08/04/2016

Web of Science
See search strategy for EMBASE, using TS= for each term, and limiting to adults by addition of following search terms:
#28 TS=adolesc*
#29 TS=child*
#30 27 NOT 28 NOT 29
