# Supplementary material for: The Effect of Technology-Mediated Diabetes Prevention Interventions on Weight: A Meta-Analysis
Source: J Med Internet Res. 2017 Mar 27;19(3):e76. doi: 10.2196/jmir.4709 (PMC5387112; doi:10.2196/jmir.4709)
Supplement: Multimedia Appendix 3 [file jmir_v19i3e76_app3.pdf]

Multimedia Appendix 3. Published studies of technology-mediated diabetes prevention interventions.

Study Cohort (Year)	Recruitment Criteria	Sample Demographics	Design & Intervention Duration	Based on DPP	Intervention Medium	Message Content & Frequency
Aguiar et al (2016) [17]	Male participants<br><br>Location: Australia<br><br>Age: 18-65<br><br>Inclusion condition(s): BMI of 25-40 and high risk for T2DM	N=53<br><br>Percent female: 0%<br><br>Age range: 20-65 years<br><br>Mean age: 52.5±9.5<br><br>Percent White: NA<br><br>Mean BMI: 32.2±3.5	RCT with technology-mediated intervention arm and standard care control<br><br>6 months	No	DVD delivered PULSE and SHED-IT weight loss and exercise program	Monitoring and logging diet and exercise using pedometer and log book. Self-monitored weight loss and waist circumference. Completed goal setting and social support tasks. Dietary and exercise messages guided by T2DM prevention guidelines and tailored to male users.
Block et al (2015) [18]	Location: California, United States<br><br>Age: 30-70<br><br>Inclusion condition(s): BMI ≥27 or ≥25 (for Asian) AND prediabetes	N=163<br><br>Percent female: 31.3%<br><br>Age range: 31-70 years<br><br>Mean age: 55.0±8.8	RCT with technology-mediated intervention arm and standard care control<br><br>6 months	Yes	Modified DPP curriculum delivered via automated interactive voice response (IVR) phone calls, automated email and text messages, website,	Tailored goal recommendations and two weekly health information topics, delivered via website, supported by mobile app. Individually tailored IVR and email delivered every two weeks. Weekly goal setting, monitoring, logging physical activity (approximately 150-300 minutes of aerobic activity per week) and decreasing added sugars and refined carbohydrates, decreasing saturated and trans fats, and increasing fruits and vegetables, reducing portion size

		Percent White: 66.9%<br><br>Mean BMI: 31.1±4.5			mobile phone application	
Sakane et al (2015) [19]	Location: Japan<br><br>Age: 20-65<br><br>Inclusion condition(s): Prediabetes	N=1240<br><br>Percent female: 17.4%<br><br>Age range: 20-65 years<br><br>Mean age: 48.9±7.8<br><br>Percent White: 0%<br><br>Mean BMI: 24.4±3.2	RCT randomly assigned participants to different technology-mediated intervention arms<br><br>12 months	No	Telephone-delivered support calls	Intervention Group-A: 3 phone calls, IG-B: 6 calls. IG-C: 10 calls during 12-month intervention. Health information, goals, and counseling to increase physical activity (10,000 steps or more or 60 minutes per week of moderate exercise), weight loss (5% reduction), increasing fiber intake, reduction in alcohol consumption
Cha et al (2014): [20]	Location: United States<br><br>Age: 18-29<br><br>Inclusion condition(s): Prediabetes	N=15<br><br>Percent female: 76.9%<br><br>Age range: 18-29 years<br><br>Mean age: 24.4±2.2	Prospective cohort<br><br>3 months	No	Mobile web platform with weekly phone calls	Bi-weekly diet and exercise assignments delivered via web and mobile application. Weekly phone counseling to review goal progress. Self-monitoring, logging physical activity and mood using external mobile applications. Content focused on health literacy (e.g. interpreting food labels), physical activity, diet diversity and vegetable intake

		Percent White: NA<br><br>Mean BMI: 41.0±7.3				
Nicklas et al (2014) [21]	Female participants<br><br>Location: Massachusetts, United States<br><br>Age: 18-45<br><br>Inclusion condition(s): GDM in most recent pregnancy and BMI	N= 36<br><br>Percent female: 100%<br><br>Mean age: 33.6±4.8<br><br>Percent White: 64.0%<br><br>Mean BMI: 30.3±6.3	RCT with technology-mediated intervention arm and standard care control<br><br>3 months	Yes	Modified DPP curriculum through 12 online videos, with internet/phone support	Weekly video, six optional modules, weekly contact with lifestyle coach via web or phone for first 12 weeks, then every other week for the second 12 weeks, and monthly thereafter. Content focused on diet (lower glycemic index, higher fiber, portion size) and physical activity (150 minutes per week) goals tracked in log books
Sepah et al (2014) [22]	Location: Recruited online<br><br>Inclusion condition(s): BMI >24 or >22 (for Asian)	N=220<br><br>Percent female: 82.7%<br><br>Mean age: 43.6±12.4<br><br>Percent White: 50.2%<br><br>Mean BMI: 36.6±7.5	Prospective cohort<br><br>12 months	Yes	DPP curriculum delivered via online group forum led by health coach	Weekly online learning modules during 4-month core phase followed by monthly online modules for 8 months during post-core phase. Content themes included monitoring, logging, changing dietary habits and increasing physical activity. Online forum allowed groups of 10-15 participants to post topics, respond, 'like' in asynchronous conversation, moderated by professional health coach.

Betzlbacher et al (2013) [23]	Location: United Kingdom<br><br>Inclusion condition(s): Prediabetes	N=55<br><br>Percent female: 43.6%<br><br>Age range: 34-85 years<br><br>Mean age: 65.3±12.6<br><br>Percent White: 91.9%<br><br>Mean BMI: 32.4±6.5	Controlled trial included control arm but assigned intervention arms by community and without randomization<br><br>6 months	No	Monthly telephone calls	Monthly phone calls providing motivational support for six-month goal attainment and positive behavior changes with diet (e.g. portion control) and lifestyle changes
Ma et al (2013) [24]	Location: California, United States<br><br>Condition: Prediabetes or MetS and BMI≥25	N=81<br><br>Percent female: 45.7%<br><br>Mean age: 51.8±9.9<br><br>Percent White: 79.0%<br><br>Mean BMI: 31.7±4.7	RCT with technology-mediated intervention arm, face-to-face intervention arm, and standard care control<br><br>3 months	Yes	DVD delivery of modified DPP curriculum called Group Lifestyle Balance, with email maintenance support, and web logging	Weekly video lessons during 3-month core phase, biweekly email reminders about self-monitoring during both core and 12-month maintenance phase, and monthly motivational messages during maintenance phase. Content encouraged goal setting, monitoring, and logging of physical activity
Piatt et al (2013): [25]	Location: Rural Pennsylvania, United States	N=113	Controlled trial with four parallel intervention	Yes	DVD delivery of the DPP curriculum,	Weekly video lessons and reminder/support phone calls. Lesson content included healthy diet choices, monitoring fat and calorie intake, physical

	Inclusion condition(s): BMI>25 and waist circumference over 40" (for males) or over 35" (for females)	Percent female: 85.0%<br><br>Mean age: 52.4±10.9<br><br>Percent White: 93.8%<br><br>Mean BMI: 36.2±7.2	arms, allocated by community and without randomization<br><br>3 months		with weekly phone support	activity, and goal setting (e.g. lose 5% of body weight and/or decrease at least one cardiovascular risk factor) with self-monitoring and logging
Piatt et al (2013): [25]	Location: Rural Pennsylvania, United States<br><br>Inclusion condition(s): BMI>25 and waist circumference over 40" (for males) or over 35" (for females)	N=101<br><br>Percent female: 88.1%<br><br>Age range: NA<br><br>Mean age: 48.7±9.7<br><br>Percent White: 99.1%<br><br>Mean BMI: 36.1±6.4	Controlled trial with four parallel intervention arms, allocated by community and without randomization<br><br>3 months	Yes	Video delivery of the DPP curriculum over the internet, with e-counseling	Weekly video lessons, email prompts for self-monitoring, physical activity, and progress updates, online counseling from preventionists and lay health coaches. Lesson content included healthy diet choices, monitoring fat and calorie intake, physical activity, and goal setting (e.g. lose 5% of body weight and/or decrease at least one cardiovascular risk factor) with self-monitoring and logging
Ramachandran et al (2013) [26]	Working men<br><br>Location: India<br><br>Age: 35-55	N=271<br><br>Percent female: 0%<br><br>Age range:	RCT with technology-mediated intervention arm and	No	Text messages	Frequency of text messages determined by participants, with median of 18 messages per month requested at baseline. Messages offered health information, motivational information and

	Inclusion condition(s): Positive family history of diabetes and BMI≥23	35-55 years<br><br>Mean age: 45.9±4.8<br><br>Percent White: 0%<br><br>Mean BMI: 25.8±3.3	standard care control<br><br>24 months			strategies for positive behavior changes in diet and exercise.
Weinstock et al (2013) [27]	Location: Upstate New York, United States<br><br>Inclusion condition(s): BMI≥30 and MetS	N=129<br><br>Percent female: 78.3%<br><br>Mean age: 50.7±13.1<br><br>Percent White: 84.5%<br><br>Mean BMI: 38.9±7.6	RCT randomly assigned participants to different technology-mediated intervention arms<br><br>12 months	Yes	Modified DPP delivered via individual phone calls	Weekly phone lessons during first 5 weeks, then monthly for 1 year. Core messages included goal-setting, self-monitoring, diet/activity modification, problem-solving.
Weinstock et al (2013) [27]	Location: Upstate New York, United States<br><br>Inclusion condition(s): BMI≥30 and MetS	N=128<br><br>Percent female: 71.9%<br><br>Mean age: 52.7±12.8<br><br>Percent White:	RCT randomly assigned participants to different technology-mediated intervention arms	Yes	Modified DPP delivered via group conference phone calls	Weekly phone lessons during first 5 weeks, then monthly for 1 year. Core messages included goal-setting, self-monitoring, diet/activity modification, problem-solving.

		85.9%<br><br>Mean BMI: 39.7±8.3	12 months			
Kramer et al (2010): [28]	Location: California, United States<br><br>Inclusion condition(s): Prediabetes or MetS and BMI≥25	N=22<br><br>Percent female: NA<br><br>Age range: NA<br><br>Mean age: 57.3±NA<br><br>Percent White: NA<br><br>Mean BMI: 32.9±6.1	Self-selected controlled trial<br><br>3 months	Yes	DVD delivery of modified DPP curriculum, with weekly phone calls	Weekly video lessons and phone calls to reinforce self-monitoring and goal attainment. Lessons focused on healthy diet, physical activity (PA), goal setting, and self-monitoring food intake and PA
Vadheim et al (2010) [29]	Location: Rural Montana, United States<br><br>Inclusion condition(s): BMI and additional risk factor (prediabetes, impaired glucose tolerance, impaired fasting glucose, high BP,	N=16<br><br>Percent female: 93.0%<br><br>Mean age: 50.0±7.0<br><br>Percent White: NA<br><br>Mean BMI: 38.7±8.0	Controlled trial<br><br>4 months	Yes	Group videoconference	Weekly sessions for 4 months, then 6 monthly sessions taught to in-person group and broadcast via video conference technology to remote intervention group. Content goals included achieving 7% weight loss and moderate physical activity for 150 minutes or more per week. Goal setting, self-monitoring, and logging of fat and calorie intake and PA

	triglycerides, cholesterol, history of GDM)					
Estabrooks and Smith-Ray (2008) [30]	Country: Colorado, United States<br><br>Condition: Prediabetes	n=39<br><br>Percent female: 71.8%<br><br>Mean age: 57.8±17.0<br><br>Percent White: 69.0%<br><br>Mean BMI: NA	RCT with technology-mediated intervention arm and standard care control<br><br>3 months	No	Weekly telephone automated interactive voice response (IVR)	Health behavior messages delivered weekly via IVR for 3 months. Messages provided counseling on physical activity and dietary health information, tips and behavior strategies
Tate et al (2003): Internet [31]	Country: Rhode Island, United States<br><br>Inclusion condition(s): BMI≥27 and at least one risk factor for T2DM	N=46<br><br>Percent female: 89.0%<br><br>Mean age: 47.3±9.5<br><br>Percent White: 89.0%<br><br>Mean BMI: 33.7±3.7	RCT randomly assigned participants to different technology-mediated intervention arms<br><br>12 months	No	Web resources	Web-based weight loss tutorial with a weekly tip and link. Weekly email with self-monitoring reminder and health information
Tate et al (2003): Internet and	Country: Rhode Island, United States	N=46	RCT randomly assigned participants to different	No	Web resources and e-counseling	Individualized messages from weight loss counselor, 5x per week in first month, weekly message in remaining 11 months of program. Web-based weight loss

eCounseling [31]	Inclusion condition(s): BMI≥27 and at least one risk factor for T2DM	Percent female: 91.0%<br><br>Mean age: 49.8±9.3<br><br>Percent White: 89.0%<br><br>Mean BMI: 32.5±3.8	technology-mediated intervention arms<br><br>12 months		tutorial with a weekly tip and link. Weekly email with self-monitoring reminder and health information
