# Supplementary material for: The Effect of Technology-Mediated Diabetes Prevention Interventions on Weight: A Meta-Analysis
Source: J Med Internet Res. 2017 Mar 27;19(3):e76. doi: 10.2196/jmir.4709 (PMC5387112; doi:10.2196/jmir.4709)
Supplement: Multimedia Appendix 4 [file jmir_v19i3e76_app4.pdf]

Multimedia Appendix 4. Change in glycaemia.

Change in Average Glycemia, Baseline to Post-Intervention						
**Study Cohort (Year): technology employed**	**Construct Measure**	**Units of Measurement**	**Mean Baseline Measure**	**Mean Post-Intervention Measure**	**p-Value**	**Based on DPP curriculum**
Aguiar et al (2016): DVD [17]	FBG	Mean±SD (mmol/l)	5.0	4.9	p=0.742	No
	A1c	Mean±SD (%)	5.8±0.5	5.4(NA)	p=0.002	
	Prediabetes prevalence	% of cohort	70%	40%	NA	
Block et al (2015): IVR, Email, Text Message, Mobile App [18]	FBG	Mean±SD (mmol/l)	6.1±0.5	5.7(NA)	p<0.001	Yes
	A1c	Mean±SD (%)	5.6±0.3	5.3(NA)	p<0.001	
	Prediabetes prevalence	% of cohort	74.2%	30%	NA	
Cha et al (2014): Internet and phone [20]	FBG	Mean±SD (mmol/l)	5.1±0.6	5.4±0.8	p=0.112	No
	A1c	Mean±SD (%)	6.0±0.5	5.6±0.5	p=0.007	
	Prediabetes prevalence	% of cohort	100%	60%	NA	
Nicklas et al (2014): Internet [21]	Diabetes incidence during intervention	% of cohort	0%	0%	NA	Yes
Sepah et al (2014): Internet [22]	A1c	Mean±SD (%)	5.98±0.1	5.6±0.1	p<0.001	Yes
Betzlbacher et al (2013): Telephone [23]	OGTT	Mean±SD (mmol/l)	9.1±0.9	7.5±2.5	p<0.001	No
	FBG	Mean±SD (mmol/l)	6.1±0.5	5.9±0.6	p<0.001	
	Prediabetes prevalence	% of cohort	100%	45.2%	NA	
	Diabetes incidence during intervention	% of cohort	0%	8.5%	NA	
Ma et al (2013): DVD and email [24]	FBG	Mean±SD (mmol/l)	5.6±0.5	5.4±0.1	p=0.2	Yes
	Diabetes incidence during intervention	% of cohort	0%	0%	NA	
Piatt et al (2013): DVD [25]	FBG	Mean±SD (mmol/l)	5.6±0.7	5.4(NA)	p=0.002	Yes
	Prediabetes prevalence	% of cohort	46%	35%	p<0.01	
Piatt et al (2013): Internet and eCounseling [25]	FBG	Mean±SD (mmol/l)	5.4±0.8	5.1(NA)	p=0.02	Yes
	Prediabetes prevalence	% of cohort	35%	29%	p>0.1	
Ramachandran et al (2013): Text message [26]	Diabetes incidence during intervention	% of cohort	0%	18%	NA	No
	FBG	Mean±SD (mmol/l)	5.97±0.5	5.7±0.6	p=0.003	Yes

Kramer et al (2010): DVD [28]	A1c	Mean±SD (%)	5.8±0.3	5.7±0.3	p=0.002

NA: not applicable or not available
OGTT: oral glucose tolerance test
FBG: fasting blood glucose
Note: Studies not listed in table did not include measurements of glycemic changes, prediabetes prevalence, and diabetes incidence.
