# Supplementary material for: The Effect of Technology-Mediated Diabetes Prevention Interventions on Weight: A Meta-Analysis
Source: J Med Internet Res. 2017 Mar 27;19(3):e76. doi: 10.2196/jmir.4709 (PMC5387112; doi:10.2196/jmir.4709)
Supplement: Multimedia Appendix 5 [file jmir_v19i3e76_app5.pdf]

Multimedia Appendix 5. Follow-up weight outcomes.

Study Cohort (Year): technology employed	Timeline of Post-Core, Maintenance, and Final Measurements	Measurement period	Weight change from baseline to end of follow up (mean±SD kg)	P value[1]	Based on DPP curriculum
Sakane et al (2015): Telephone [19]	Post-core: 12 months<br>Maintenance: NA<br>Final Measurement: 48 months	Measured from baseline to 48 months	-1.1±3.4	p<0.05	No
Sepah et al (2014): Internet [22]	Post-core: 4 months<br>Maintenance phase duration: 8 months<br>Final Measurement: 12 months	Measuring from baseline to 12 months	-4.85±0.5	p<0.0001	Yes
Betzlbacher et al (2013 and f/u study): Telephone [23]	Post-core: 6 months<br>Maintenance: NA<br>Final Measurement: 18 months	Measured from baseline to 18 months	-2.8±4.9	P<0.01	No
Ma et al (2013): DVD [24]	Post-core: 3 months<br>Maintenance phase: 12 months<br>Final Measurement: 15 months	Measured from baseline to 15 months	-4.5±0.9	P=0.02	Yes
Piatt et al (2013 and f/u study): DVD [25]	Post-core: 3 months<br>Maintenance phase: NA<br>Final Measurement: 18 months	Measured from baseline to 18 months	-4.5±NA	p<0.0001	Yes
Piatt et al (2013 and f/u study): Internet [25]	Post-core: 3 months<br>Maintenance phase: NA<br>Final Measurement: 18	Measured from baseline to 18 months	-5.2±NA	p<0.0001	Yes

	months				
Weinstock et al (2013 and f/u study): Individual telephone [27]	Post-core: 12 months<br>Maintenance phase: 12 months<br>Final Measurement: 36 months	Measured from baseline to 36 months	-2.4±16.3	p≤0.05	Yes
Weinstock et al (2013 and f/u study): Group telephone [27]	Post-core: 12 months<br>Maintenance phase: 12 months<br>Final Measurement: 36 months	Measured from baseline to 36 months	-6.4±15.6	p≤0.001	Yes

[1]p-values reported only for those studies where the data is available
NA: not applicable or not available
Note: Studies not listed in table did not include measurements of glycemic changes, prediabetes prevalence, and diabetes incidence.
